# Synthesis and Crystal Structure Characterization of Zinc (II) Tetronic Acid Complexes

**DOI:** 10.1155/2010/651652

**Published:** 2010-11-04

**Authors:** K. C. Prousis, G. Athanasellis, V. Stefanou, D. Matiadis, E. Kokalari, O. Igglessi-Markopoulou, V. McKee, J. Markopoulos

**Affiliations:** ^1^Laboratory of Organic Chemistry, School of Chemical Engineering, National Technical University of Athens, 15773, Athens, Greece; ^2^Laboratory of Inorganic Chemistry, Department of Chemistry, University of Athens, Panepistimiopolis, 15771 Athens, Greece; ^3^Chemistry Department, University of Loughborough, Leicestershire LE11 3TU, UK

## Abstract

The synthesis and characterization of two new tetronic acid zinc (II) complexes of the empirical formulae [Zn(L–H)_2_(H_2_O)_2_] (**1**) and [Zn(L–H)_2_(H_2_O)(MeOH)]H_2_O (**2**) found within the same crystal are reported. The zinc ions bind through alkoxide and carbonyl groups of the ligand 3-methoxycarbonyl-5-phenyl tetronic acid (LH) as indicated by ^1^H NMR and X-ray crystallographic studies. These complexes promote intra- and intermolecular interactions, such as hydrogen bonding and *π* stacking, giving place to the formation of molecular aggregates.

## 1. Introduction

**Scheme 1:**
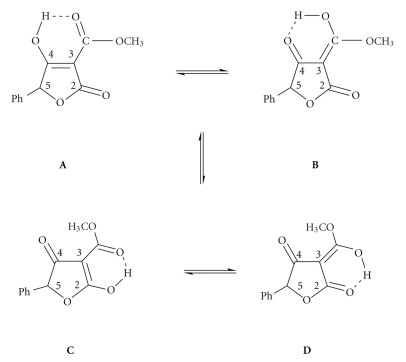
3-Methoxycarbonyl-5-phenyltetronic acid.

Tetronic acid derivatives, tetrahydrofurane-2,4-diones (Scheme 1), represent an important class of oxygen 5-membered heterocyclic compounds containing the *β*-diketonate moiety [1]. A classical example of this type of structure is the molecule of ascorbic acid (vitamin C). These compounds are structural motifs in many natural products [2] exhibiting a wide range of biological activities including antibiotic, antiviral, antineoplastic, and anticoagulant activity [3]. Recently, 3-carboxamide tetronic acids were investigated as inhibitors of undecaprenyl pyrophosphate synthase (UPPS) for use as antimicrobial agents [4] while compounds which have been isolated from natural products and exhibit such activity are tetronasin [5], RK-682 (3-alkanoyl-5-hydroxymethyl tetronic acid) [6, 7], and the well-known family of compounds named vulpinic acids [8, 9]. In addition, several nonnatural 3-functionalized tetronic acids have been reported as antioxidant and anti-inflammatory compounds [6].

The interesting biological and structural diversity of these compounds has raised the attention of chemists and biologists by reason of their challenging structural complexity and their high affinity to various sorts of “biological effectors” ranging from simple “metal cations” to complex enzymes [10].

Tetronates, containing the *β*-diketo acid pharmacophoric motif, could be involved as model ligands for binding with divalent metal ions, which are critical cofactors at the enzyme catalytic site [11]. 

The coordination chemistry of zinc (II) *β*-diketonates has been extensively explored as a model compound for the understanding of the chemistry of zinc (II) complexes, where a metal-oxygen bond is formed. The most important and best role of zinc is as a structural cofactor in metalloproteins. Even more, the metal *β*-diketonates including zinc (II) complexes are a promising class of compounds as inhibitors of HIV-1 and of integrase (IN), since the active site of the enzyme interacts with the metal diketonates [12]. It is well known that zinc (II) activates essential enzymes such as carboxypeptidase or alcohol dehydrogenase providing a complex with the active site. Also, the zinc ion is an important structural component in proteins in nucleic acid binding (“zinc-fingers” transcription factors) [13, 14]. Meanwhile, zinc (II) ions are directly associated with the regulation of gene expression through metalloregulatory proteins. In addition, zinc (II) ions are also present in most DNA and RNA polymerases [15]. Moreover, the metalloneurochemistry of zinc (II) is of substantial current interest; zinc and d-block metals are emerging as significant players in neurophysiology, aging, and neuropathology [16]. Zinc is the second most abundant d-block metal ion in the human brain, and its distribution varies with relatively high concentrations found in the hippocampus [17]. It is interesting to point out that the tetracoordinated zinc (II) complexes are a promising class of versatile synthons to supramolecular architectures, since the driving force to aggregation occurs through the metal center [18], hydrogen bonds, and *π*-stacking [19, 20]. Recent development on the design and synthesis of zinc (II) fluorescent probes including chemosensors with fluorogenic agents and biosensors has been done and referred [15].

In the course of our research program on transition metal complexes with heterocyclic scaffolds containing the *β*-diketo functionality [21–23], we reported in a recent work the coordination model of tetronic acids with Cu (II) and Co (II) ions [10, 24].

In the present paper, we report a detailed investigation of new zinc (II) complexes, [Zn(L–H)_2_(H_2_O)_2_] (**1**) and [Zn(L–H)_2_(H_2_O)(MeOH)]H_2_O (**2**), involving the *β*-diketo-tetronic acids as “model ligands”. The structure and “supramolecular” arrangements of the isolated complexes have been investigated by single crystal X-ray crystallography.

The “ligand molecules” have a proton adjacent to the carbonyl group and consequently can exhibit enol-enol tautomerism as shown in Scheme 1.

## 2. Experimental

### 2.1. Materials and Instruments

All reagents were purchased from Aldrich, Fluka, and Acros and were used without further purification. Dry THF was distilled from Na/Ph_2_CO. Melting points were determined on a Gallenkamp MFB-595 melting point apparatus and are uncorrected. IR spectra were recorded on a Jasco 4200 FTIR spectrometer. NMR spectra were recorded on a Varian Gemini-2000 300 MHz spectrometer operating at 300 MHz (^1^H) and 75 MHz (^13^C). Chemical shifts are reported in ppm relative to DMSO-d_6_ (^1^H: *δ* = 2.50, ^13^C: *δ* = 77.16). J values are given in Hz.

The X-ray crystals were obtained from a solution of methanol diffused with diethyl ether. Data were collected at 150(2) K on a beamline 9.8 of the Synchrotron Radiation Source, Daresbury using a very thin plate but the mosaicity was still high. The structure was solved by direct methods and refined on *F* ^2^ using all the reflections. All the nonhydrogen atoms were refined using anisotropic atomic displacement parameters and hydrogen atoms were inserted at calculated positions using a riding model. 

### 2.2. Compound Preparation

#### 2.2.1. 3-Methoxycarbonyl-5-Phenyl Tetronic Acid

The ligand was prepared, purified and characterized following our research group's method [25–27].

White solid (78%), m.p. 188-189°C; (Anal. Found: C, 61.60; H, 4.21. Calc. for C_12_H_10_O_5_: C, 61.54; H, 4.27%); IR (KBr) *ν*_max _/cm^−1^ (C=O) 1759, 1716, (C=C) 1610. ^1^H-NMR *δ*_H_ (ppm, DMSO-d_6_): 3.64 (3H, s, COOCH_3_), 5.67 (1H, s, CH ring), 7.28–7.42 (5H, m, aromatic protons), 8.65 (enolic OH). ^13^C-NMR *δ*_C_ (ppm, DMSO-d_6_): 50.7(COOCH_3_),78.8 (C-5), 90.6 (C-3), 127.3/128.8/129.1/135.1 (aromatic carbons), 162.1 (C-6), 69.3 (C-2), 186.9 (C-4).

#### 2.2.2. General Method of Preparation of the Complexes

The synthesis of the complexes was accomplished by mixing the ligand (2 eq) with zinc acetate [Zn(OCOCH_3_)_2_ · 2H_2_O, 1 eq.] in methanol and refluxing the solution for about 2 hours. The resulting solution was evaporated up to a small volume and the precipitate deposited was collected by filtration, washed with cold methanol, diethyl ether and dried in vacuo over P_2_O_5._ The structural elucidation of the complex isolated was accomplished by IR and NMR spectroscopy as well as X-ray Crystallography. X-ray quality crystals were obtained from a solution of methanol diffused with ether. 

White solid (67%); (Anal. Found: C, 50.53; H, 4.05; Calc. For Zn_2_C_48.5_H_47_O_25_: C, 50.34; H, 4.06%). IR (KBr) *ν*_max _/cm^−1^ (OH) 3654 w, 3188 br, (C=O and C=C) 1732 s, 1650 s, 1575 s, 1480 s, 1419 m, (Zn-O) 472 w, 428 w. ^1^H-NMR *δ*_H_ (ppm, DMSO-d_6_): 3.59 (s, 3H, COOCH_3_), 5.42 (s, 1H), 7.26–7.35 (m, 5H, aromatic protons), 3.16–3.18 (d, CH_3_ methanol), 4.08–4.10 (q, OH, methanol).^ 13^C-NMR *δ*_C_ (ppm, DMSO-d_6_): 48.6 (COOCH_3_), 80.4 (C-5), 84.3 (C-3), 126.5/128.2/128.3/136.7 (aromatic carbons), 168.5 (C-6), 172.3 (C-2), 195.1 (C-4).

### 2.3. X-Ray Crystallography

Parameters for data collection and refinement are summarized in Table 1.

## 3. Results and Discussion

### 3.1. Synthetic Comments, NMR and IR Spectra

The tetronic acid ligand precursor containing the 5-phenyl group (Scheme 1) was synthesized according to a new methodology presented by our group [25–27]. The methodology which was chosen to be followed has the advantages of small reaction times and isolation of products in good yields and satisfactory purity. Moreover, it is stereoselective; therefore, it could be followed in case of other chiral tetronic acids. This strategy is very helpful in situations where chiral molecules are to be used as ligands in coordination chemistry. The reaction of 3-methoxycarbonyl-5-phenyl tetronic acid with zinc (II) acetate gave the bis ligand Zn (II) complexes. The new complexes were characterized by NMR and IR Spectroscopy, elemental analysis, and X-ray crystallography. The ^1^H-NMR spectrum in DMSO-*d*_6_ of the complex confirms the formation of metal-oxygen bonds since the signal of the acidic proton of the ligand (around 8.65 ppm) is absent in the metal complex.

### 3.2. Description of the Structures

**Figure 1:**
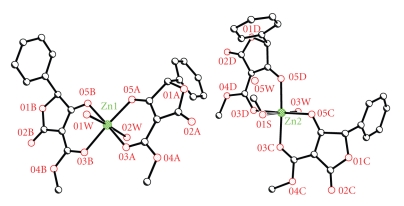
X-ray structure of the two independent complexes in the asymmetric unit of the metal complex, Zn1 and Zn2.

**Figure 2:**
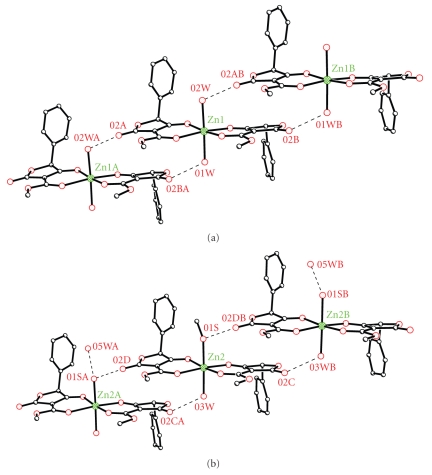
X-ray structure of the metal complexes showing the hydrogen bonds.

The molecular structures of complexes Zn1 and Zn2 are shown in Figures 1 and 2, whereas selected bond lengths and angles as well the hydrogen bond lengths are listed in Tables 2 and 3 consequently. The structure was solved in space group C2 and refined as a racemic twin (BASF 0.17) with two similar molecules in the asymmetric unit. The zinc ions are six-coordinate, each coordinated to two monodeprotonated, bidentate ligands which bind through alkoxide and carbonyl groups and are *cis* to one another. The coordination sphere about Zn1 is completed by two coordinated water molecules. In the second molecule (containing Zn2), one of the axial sites is disordered and has been modeled as 50 : 50 disorder between methanol and water (with the oxygen atoms on the same site), with a further uncoordinated water molecule hydrogen bonded to the partial occupancy water ligand (Figure 1). 

According to the data provided by X-ray Crystallography Analysis, the complex comprises of three different subcomplexes: (a) the Zn(L–H)_2_(H_2_O)_2_ which is the molecule 1 centered on Zn1, (b) the [Zn(L–H)_2_(H_2_O)]H_2_O which represents the 50% percentage of molecule 2 centered on Zn_2_, and (c) the Zn(L–H)_2_(H_2_O)(MeOH) which represents the other 50% percentage of molecule 2 centered on Zn_2_. If we add appropriately the above three complexes (100% of complex (a) + 50% of complex (b) + 50% of complex (c)), then the molecular formula found is Zn_2_(L–H)_4_(H_2_O)_4.5_(MeOH)_0.5_ or Zn_2_C_48.5_H_47_O_25_.

**Figure 3:**
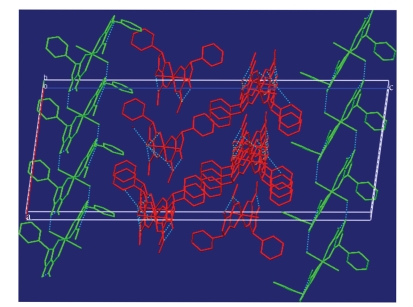
Zn1 chains (red) and Zn2 chains (green).

Hydrogen bonding between the coordinated water (or methanol) molecules and the uncoordinated carbonyl groups of neighboring molecules link the complexes into one-dimensional chains. Each molecule gives rise to an independent chain, those comprising symmetry equivalents of Zn2 run parallel to the b axis, while those composed of symmetry equivalents of Zn1 lie along the ab diagonal (Figures 2 and 3). 

## 4. Conclusions

The use of 3-methoxycarbonyl-5-phenyl tetronic acid in reaction with Zn(OCOCH_3_)_2_ · 2H_2_O has yielded two mononuclear [Zn(L–H)_2_(H_2_O)_2_] (**1**) and [Zn(L–H)_2_(H_2_O)(MeOH)]H_2_O (**2**), complex compounds. The crystal structure of the complexes shows the existence of two independent molecules in the asymmetric unit in the crystal moiety. The complexation is achieved through the oxygens of the alkoxide groups and the carbonyl functionalities. Hydrogen bonding between water or methanol molecules of the unit gives rise to two one-dimensional independent chains. These models are promising systems for the development of new supramolecular architectures. Design and results on the synthesis of zinc (II) fluorescent probes with chemosensors heterocyclic molecules containing the *β*-diketo functionality will be reported in due course.

## Figures and Tables

**Table 1:** Crystal data, structure refinement for [Zn(L–H)_2_(H_2_O)_2_][Zn(L–H)_2_(H_2_O)(MeOH)]H_2_O.

Empirical formula	C_24.25_H_23_O_12.25_Zn
Formula weight	575.80
Temperature	150(2) K
Wavelength	0.68840 Å
Crystal system	Monoclinic
Space group	C2
Unit cell dimensions	a = 15.274(7) Å, *α* = 90°
	b = 8.812(4) Å, *β* = 97.664(4)°
	c = 39.525(18) Å, *γ* = 90°.
Volume	5272(4) Å^3^
Z	8
Density (calculated)	1.451 Mg/m^3^
Absorption coefficient	0.994 mm^−1^
F(000)	2372
Crystal size	0.16 × 0.10 × 0.02 mm^3^
Crystal description	colourless plate
Theta range for data collection	2.59 to 25.00°.
Index ranges	−18≤*h*≤18, −10≤*k*≤10, −48≤l≤48
Reflections collected	14618
Independent reflections	8420 [*R*(int) = 0.0388]
Completeness to theta = 25.00°	83.4 %
Absorption correction	Semi-empirical from equivalents
Max. and min. transmission	0.9804 and 0.8571
Refinement method	Full-matrix least-squares on F^2^
Data/restraints/parameters	8420/696/704
Goodness-of-fit on *F* ^2^	1.132
Final *R* indices [I > 2 sigma (I)]	*R*_1_ = 0.0895, *w* *R*_2_ = 0.2373
*R* indices (all data)	*R*_1_ = 0.0928, *w* *R*_2_ = 0.2396
Absolute structure parameter	0.17(2)
Largest diff. peak and hole	1.905 and −0.959 e.Å^−3^

**Table 2:** Selected bond lengths [Å] and angles [°] for [Zn(L–H)_2_(H_2_O)_2_][Zn(L–H)_2_(H_2_O)(MeOH)]H_2_O.

Bond	Bond length	Bond	Bond length
Zn(1)–O(5A)	2.062(7)	Zn(2)–O(5C)	2.069(7)
Zn(1)–O(5B)	2.062(7)	Zn(2)–O(3W)	2.079(8)
Zn(1)–O(1W)	2.104(7)	Zn(2)–O(1S)	2.089(7)
Zn(1)–O(2W)	2.109(8)	Zn(2)–O(5D)	2.103(7)
Zn(1)–O(3B)	2.115(7)	Zn(2)–O(3D)	2.116(8)
Zn(1)–O(3A)	2.154(7)	Zn(2)–O(3C)	2.139(7)
O(1A)–C(1A)	1.394(12)	O(1C)–C(1C)	1.414(14)
O(1A)–C(6A)	1.457(11)	O(1C)–C(6C)	1.443(12)
C(1A)–O(2A)	1.191(12)	C(1C)–O(2C)	1.177(13)
C(1A)–C(2A)	1.444(13)	C(1C)–C(2C)	1.437(13)
C(2A)–C(5A)	1.391(12)	C(2C)–C(5C)	1.390(14)
C(2A)–C(3A)	1.449(12)	C(2C)–C(3C)	1.426(15)
C(3A)–O(3A)	1.215(12)	O(3C)–C(3C)	1.236(13)
C(3A)–O(4A)	1.337(11)	C(3C)–O(4C)	1.339(13)
O(4A)–C(4A)	1.444(13)	O(4C)–C(4C)	1.535(16)
C(5A)–O(5A)	1.258(11)	C(5C)–O(5C)	1.259(12)
C(5A)–C(6A)	1.530(12)	C(5C)–C(6C)	1.551(16)
C(6A)–C(7A)	1.509(14)	C(6C)–C(7C)	1.494(17)
C(7A)–C(8A)	1.371(15)	C(7C)–C(8C)	1.386(18)
C(7A)–C(12A)	1.393(14)	C(7C)–C(12C)	1.385(19)
O(5A)–Zn(1)–O(5B)	90.7 (3)	O(5C)–Zn(2)–O(3W)	87.1(3)
O(5A)–Zn(1)–O(1W)	93.9 (3)	O(5C)–Zn(2)–O(1S)	94.7(3)
O(5B)–Zn(1)–O(1W)	87.5 (3)	O(3W)–Zn(2)–O(1S)	177.9(4)
O(5A)–Zn(1)–O(2W)	88.3 (3)	O(5C)–Zn(2)–O(5D)	90.4(3)
O(5B)–Zn(1)–O(2W)	96.4(3)	O(3W)–Zn(2)–O(5D)	93.1(3)
O(1W)–Zn(1)–O(2W)	175.4(3)	O(1S)–Zn(2)–O(5D)	87.9(3)
O(5A)–Zn(1)–O(3B)	174.5(3)	O(5C)–Zn(2)–O(3D)	174.2(3)
O(5B)–Zn(1)–O(3B)	90.3(2)	O(3W)–Zn(2)–O(3D)	87.2(3)
O(1W)–Zn(1)–O(3B)	91.5(3)	O(1S)–Zn(2)–O(3D)	91.0(3)
O(2W)–Zn(1)–O(3B)	86.2(3)	O(5D)–Zn(2)–O(3D)	88.8(3)
O(5A)–Zn(1)–O(3A)	89.6(3)	O(5C)–Zn(2)–O(3C)	90.0(3)
O(5B)–Zn(1)–O(3A)	173.1(3)	O(3W)–Zn(2)–O(3C)	92.6(3)
O(1W)–Zn(1)–O(3A)	85.5(3)	O(1S)–Zn(2)–O(3C)	86.4(3)
O(2W)–Zn(1)–O(3A)	90.5(3)	O(5D)–Zn(2)–O(3C)	174.3(3)
O(3B)–Zn(1)–O(3A)	90.0(3)	O(3D)–Zn(2)–O(3C)	91.4(3)

**Table 3:** Hydrogen bonds for [Zn(L–H)_2_(H_2_O)_2_][Zn(L–H)_2_(H_2_O)(MeOH)]H_2_O [Å and °].

D–H⋯A	d(D–H)	d(H*⋯*A)	d(D*⋯*A)	<(DHA)
O(1S)–H(1S)*⋯*O(2D)#1	0.85	1.80	2.651(11)	179.0
O(4W)–H(4WA)*⋯*O(2D)#1	0.84	1.83	2.651(11)	162.3
O(4W)–H(4WB)*⋯*O(5W)	0.89	2.39	2.77(3)	106.0
O(1W)–H(1WA)*⋯*O(2B)#2	0.84(2)	1.84(3)	2.677(10)	170.0(13)
O(2W)–H(2WA)*⋯*O(2A)#3	0.84(2)	1.82(2)	2.685(10)	176.0(13)
O(3W)–H(3WA)*⋯*O(2C)#4	0.83(2)	1.88(4)	2.698(11)	168.0(12)
